# Prevalence of computer vision syndrome: a systematic review and meta-analysis

**DOI:** 10.1038/s41598-023-28750-6

**Published:** 2023-01-31

**Authors:** Etsay Woldu Anbesu, Asamene Kelelom Lema

**Affiliations:** 1 Department of Public Health, College of Medical and Health Sciences, Samara University, Semera, Ethiopia; 2 Department of Computer Science, College of Engineering and Technology, Samara University, Semera, Ethiopia

**Keywords:** Health care, Medical research

## Abstract

Although computer vision syndromes are becoming a major public health concern, less emphasis is given to them, particularly in developing countries. There are primary studies on different continents; however, there are inconsistent findings in prevalence among the primary studies. Therefore, this systematic review and meta-analysis aimed to estimate the pooled prevalence of computer vision syndrome. In this study, the review was developed using the Preferred Reporting Items for Systematic Reviews and Meta-Analyses guidelines. Online electronic databases, including PubMed/Medline, CINAHL, and Google Scholar, were used to retrieve published and unpublished studies. The study was conducted from December 1 to April 9/2022. Study selection, quality assessment, and data extraction were performed independently by two authors. Quality assessment of the studies was performed using the Joanna Briggs Institute Meta-Analysis of Statistics Assessment and Review Instrument tool. Heterogeneity was assessed using the statistical test I^2^. STATA 14 software was used for statistical analysis. A total of 7,35 studies were retrieved, and 45 studies were included in the final meta-analysis. The pooled prevalence of computer vision syndrome was 66% (95% CI: 59, 74). Subgroup analysis based on country was highest in Pakistan (97%, 95% CI: 96, 98) and lowest in Japan (12%, 95% CI: 9, 15). Subgroup analysis based on country showed that studies in Saudi Arabia (I^2^ = 99.41%, *p* value < 0.001), Ethiopia (I^2^ = 72.6%, *p* value < 0.001), and India (I^2^ = 98.04%, *p* value < 0.001) had significant heterogeneity. In the sensitivity analysis, no single study unduly influenced the overall effect estimate. Nearly two in three participants had computer vision syndrome. Thus, preventive practice strategic activities for computer vision syndrome are important interventions.

## Introduction

Computer vision syndrome (CVS) is defined as “a complex of eye and vision problems related to near work experienced during computer use”^1^. Visual fatigue (VF) and digital eye strain (DES) terms are also used for CVS, reflecting the different digital devices related to potential health problems^2^. Symptoms related to CVS can be classified as visual, ocular, and extraocular symptoms^3^. Visual symptoms include blurred vision, visual fatigue or discomfort, and diplopia^4–7^. Ocular symptoms include dry eye disease, redness, eye strain, and irritation^1,8,9^. Extraocular symptoms include headache and shoulder, neck, and back pain^3,4,10–14^.

Individuals spend more time on electronic devices such as computers, laptops, smartphones, tablets, and e-readers, which contribute to CVS^15^. Children are also affected in CVS, as they spend many hours using electronic devices for schoolwork, playing video games, and sending and receiving text messages^15^. However, the use of these devices even for 3 h/day can lead to the development of CVS^3^.

The massive growth of digital devices has become an integral part of daily life, and millions of individuals of all ages are at risk of CVS^16–18^. In developed nations, engagement with digital devices has increased substantially in recent years across all age groups^19–22^. Moreover, digital device use has increased in developing countries, resulting in a high burden of CVS due to low accessibility, low utilization of personal protective equipment, and limited break time while using electronic devices. CVS is a major public health problem leading to occupational hazard, an increased error rate, impaired visual abilities, reduced productivity, and low job satisfaction^23,24^.

A review of the literature showed that factors associated with CVS can be classified as personal factors, which include poor sitting position, inappropriate eye-to-screen distance, insufficient working procedures, improper viewing angle and distances, age, medical diseases, and long duration of computer usage. The environment and computer factors such as improper workstations, poor lighting, contrast, and resolution rooms, slow refresh rate, glare of the display, excessive screen brightness, and imbalance of light between the computer screen and surrounding working room^5,10,25–28^.

Modern digital technology markedly influences the daily activities and lifestyles of people^4,7^. CVS has an effect on reduced productivity and visual and musculoskeletal impairment and a negative impact on cadiac rhythms and sleep patterns^4,7,13,29,30^. Although CVS is becoming a major public health problem, less emphasis is given, particularly in developing countries. There are primary studies on different continents; however, there are inconsistent findings in prevalence among the primary studies. Therefore, this systematic review aimed to estimate the pooled prevalence of computer vision syndrome.

## Methods

### Protocol and registration

This systematic review and meta-analysis was registered on PROSPERO with registration number CRD42022325167. Available at: https://www.crd.york.ac.uk/prospero/#myprospero.

### Search strategies

The systematic review was developed using the Preferred Reporting Items for Systematic Reviews and Meta-Analyses (PRISMA) guidelines^31^, and the review procedure was reported using the PRISMA-P 2009 checklist^32^ *(*supplementary file 1*).* Published and unpublished studies were searched in databases such as Medline/PubMed, CINAHL, and Google Scholar from December 1 to April 9/2022. MeSH terms and entry terms were used to search studies from databases, and modifications were made based on the type of database *(*supplementary file 2*).*

### Eligibility criteria

#### Inclusion criteria

- The following criteria were considered to include studies:

Study area.

- Anywhere

Study scope.

- Studies that report the prevalence of CVS and its associated factors
- Studies that report the prevalence of CVS
- “Both community- and facility-based studies”
- Quantitative results, if the study reported both qualitative and quantitative results

Study design.

- Observational study designs, including cross-sectional and cohort study designs

Language.

- English

Population.

- All population groups

Publication year.

- No restriction

### Exclusion criteria

Studies were excluded if:

- Other than English
- Studies that did not report specific outcomes (prevalence) of CVS
- No full-text article following email contact to the corresponding authors
- Qualitative studies
- Letters, conference abstracts, case reports, and reviews,

### CoCoPop/PEO

*Condition*: computer vision syndrome.

*Context*: worldwide.

*Population*: All population groups.

*Outcome/context*: *The primary outcome of the study was the pooled prevalence of CVS. The* prevalence of CVS was considered when the studies reported the overall prevalence of CVS or either of CVS syndromes (blurred vision, eye strain/fatigue, discomfort, diplopia, dry eye disease, redness, irritation, headache, shoulder, neck, and back pain) in the primary studies.

### Study selection

Endnote reference manager software^33^ was used to organize and remove duplicates, irrelevant titles, and abstracts. Duplicate studies were removed. An assessment of studies using the title and abstract was performed, and irrelevant titles and abstracts were removed. Study selection was performed independently by two reviewers (EW and AK). The selection procedures of the studies were presented using a PRISMA diagram*.*

### Quality assessment

A full-text review of studies was performed before the inclusion of studies in the final meta-analysis using “The Joanna Briggs Institute Meta-Analysis of Statistics Assessment and Review Instrument (JBI-MAStARI)”^34^ quality appraisal tool. The components of quality assessment include study setting, outcome and explanatory variable measurements, clear inclusion criteria, measurement criteria used, participants’ description**, **and valid statistical analysis*.* Independent quality assessment of the studies was reviewed by EW and AK, and studies with a quality score of 50% and above were included in the final systematic review and meta-analysis. Disagreement during quality assessment among reviewers was resolved with discussion. In addition, cross-referencing of the included articles was performed.

### Data extraction

Independent data extraction was performed by the authors (EW and AK) using a pilot-tested data extraction Microsoft Office Excel sheet. The data extraction sheet elements included publication year, authors’ names, study design, country, sample size, response rate, prevalence and study subjects. Discrepancies were resolved by discussion between the authors (EW and AK). Contact with the corresponding authors of the studies was made for incomplete data, and the study was excluded if there was no response.

### Data analysis

The extracted Excel data were imported into STATA version 14 for analysis. A narrative description and summary characteristics of the included studies were reported in tables and graphs. A random-effects model meta-analysis^35^ was used to estimate the overall effect size, and the results were presented using a forest plot.

The heterogeneity of studies was assessed by the I^2^ statistic^36^. I^2^ statistics of 25, 50 and 75% showed low, moderate and high heterogeneity, respectively, with *p* < 0.05. Publication bias was assessed using visual observation of the funnel plot^37^ and Egger’s test at *p* < 0.05^38^. To identify the sources of heterogeneity among the studies, subgroup analysis and meta-regression^39^ were performed based on country and sample size. Moreover, sensitivity analysis was performed to assess the effect of the study on the overall effect size.

### Results

**Figure 1:**
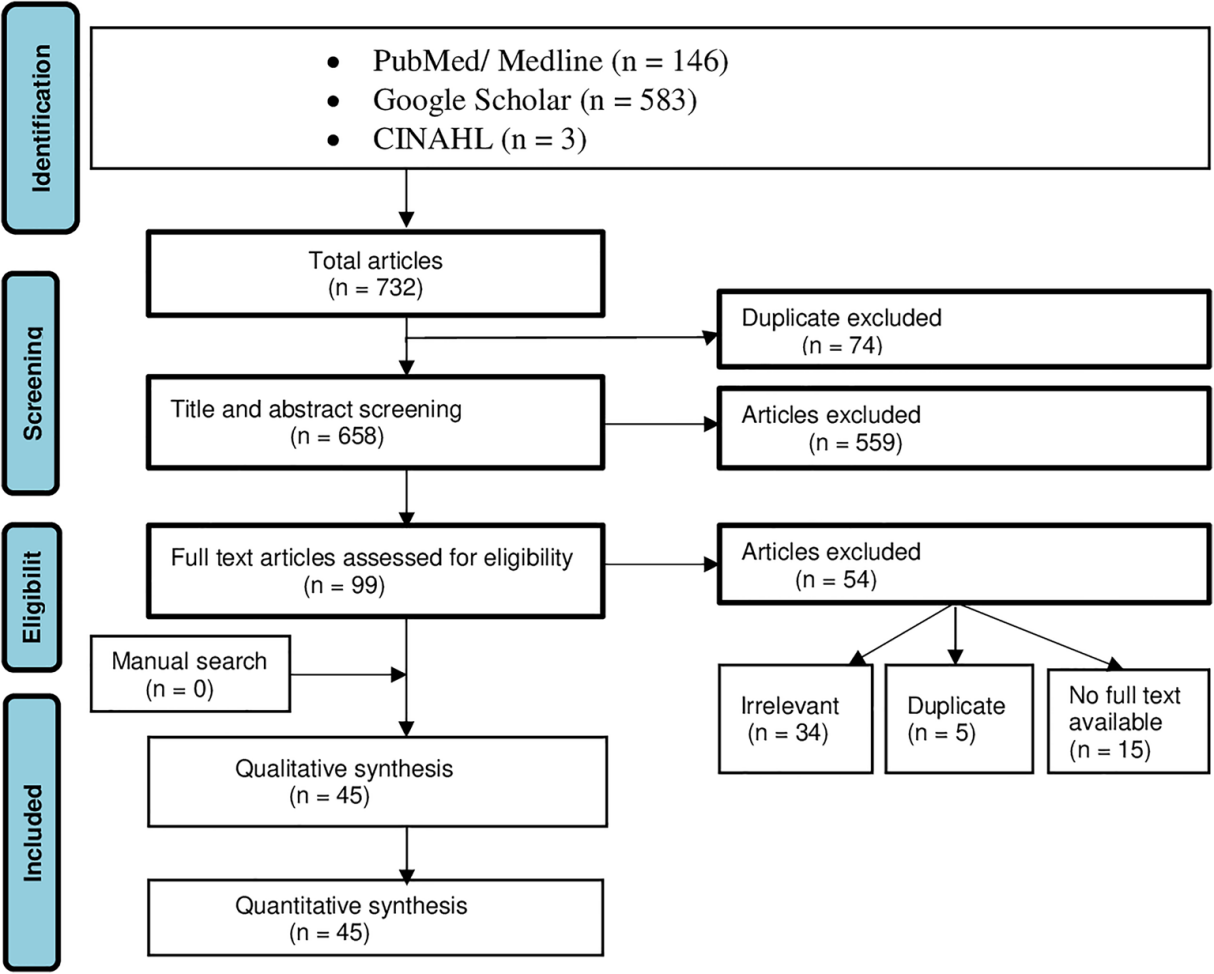
PRISMA flow diagram studies screening, and selection on computer vision syndrome, 2022.

A total of 735 articles were retrieved using electronic database searches: PubMed, Google Scholar, and CINHAL. Seventy-seven articles were excluded due to duplication, and 559 articles were excluded because they were not related to the title and abstract. Ninety-nine full-text articles were assessed for quality eligibility, and 57 articles were excluded based on the quality appraisal tool because they were irrelevant, had no full text available, or were duplicates. Three articles were identified through a cross-reference search of the included studies. Finally, 45 articles were included in the systematic review and meta-analysis (Fig. 1).

### Characteristics of the included studies

A total of 45 cross-sectional studies with 17,526 sample sizes were included in this systematic review and meta-analysis: four studies in Saudi Arabia^40–43^, two studies in Nigeria^44,45^, three studies in Ghana^46–48^, four studies in Pakistan^49–52^, three studies in Spain^53–55^, seven studies in Ethiopia^56–62^, one study in Jordan^63^, two studies in China^64,65^, one study in Iran^66^, three studies in Egypt^67–69^, eight studies in India^18,70–76^, one study in Nepal^77^, one study in Sri Lanka^29^, two studies in Brazil^78,79^, one study in Beirut^80^, one study in Japan^81^, and one study in Thailand^82^. The sample size ranged from 74 in China^64^ to 2210 in Sri Lanka^29^ (Table 1).

**Table 1:** Characteristics of included studies in the meta-analysis of computer vision syndrome, 2022.

Author/s/year (reference)	Country	Study design	Sample size	Response rate (%)	Prevalence (%)	Study subjects
Abudawood GA, et al. ^41^	Saudi Arabia	Cross sectional	587	100	95.1	Students
Agbonlahor O. et al.^44^	Nigeria	Cross sectional	215	84	65.1	Government employ
Akowuah PK, et al.^83^	Ghana	Cross sectional	362	92.5	64.4	Students
Al Dandan O, et al.^42^	Saudi Arabia	Cross sectional	198	75.3	50.5	Radiologists
Al Subaie M, et al.^43^	Saudi Arabia	Cross sectional	416	100	43.5	Population ≥ 15 years
Arshad S, et al.^84^	Pakistan	Cross sectional	320	100	58.1	Students
Artime‐Ríos E, et al.2021 ^53^	Spain	Cross sectional	622	-	56.7	Health workers
Boadi-Kusi SB, et al.^48^	Ghana	Cross sectional	139	86.9	71.2	Bank workers
Boadi-Kusi SB, et al.^47^	Ghana	Cross sectional	200	65	51.5	University staff
Cantó‐Sancho N, et al.^54^	Spain	Cross sectional	244	100	76.6	Students
Derbew H, et al.^56^	Ethiopia	Cross sectional	351	98	74.6	Bank workers
Dessie A, et al.^57^	Ethiopia	Cross sectional	607	93.1	69.5	Government employ
Gammoh Y. et al.^63^	Jordan	Cross sectional	382	92	94.5	Students
Gondol BN, et al.^58^	Ethiopia	Cross sectional	272	100	81.3	Government employ
Han CC, et al.^65^	China	Cross sectional	1469	97.9	57.04	Students
Hashemi H, et al.^66^	Iran	Cross sectional	1040	97.2	49.4	Students
Kamal NN, et al.^67^	Egypt	Cross sectional	218	96.3	84.8	Bank workers
Lakachew Assefa N. et al.^59^	Ethiopia	Cross sectional	304	98.2	73.03	Bank workers
Lemma MG. et al.^60^	Ethiopia	Cross sectional	455	93	68.8	Secretaries
Lemma MT,et al.^61^	Ethiopia	Cross sectional	217	96.8	75.6	Secretaries
Logaraj M, et al.^70^	India	Cross sectional	215	100	81.8	Students
Mansoori N, et al.^50^	Pakistan	Cross sectional	150	100	28	students
Mohan A, et al.^71^	India	Cross sectional	217	83.14	50.2	Children
NAGWA E, et al.^68^	Egypt	Cross sectional	260	100	75	Students
Noreen K, et al.^52^	Pakistan	Cross sectional	326	95.04	98.7	Students
Noreen K, et al.^51^	Pakistan	Cross sectional	198	86.5	67.2	Students
Nwankwo B, et al.^45^	Nigeria	Cross sectional	153	100	54.2	Students
Poudel S, et al.^77^	Nepal	Cross sectional	263	94.9	82.5	IT office workers
Rafeeq U, et al.^72^	India	Cross sectional	120	100	69.2	≥ 12 years old population
Ranasinghe P, et al.^85^	Serilanka	Cross sectional	2210	88.4	67.4	Computer office workers
Ranganatha SC, et al.^73^	India	Cross sectional	150	100	86.7	Computer sciences students
Rathore D. , et al.^74^	India	Cross sectional	150	100	75.3	Computer users
Sa EC, et al.^78^	Brazil	Cross sectional	476	89.6	54.6	Call center
Sánchez-Brau M, et al.^55^	Spain	Cross sectional	109	95.6	74.3	Visual display workers
Sawaya RI, et al.^80^	Beirut	Cross sectional	457	73.5	67.8	Students
Singh H, et al.^18^	India	Cross sectional	192	96	51.6	Students
Tiwari RR, et al.^75^	India	Cross sectional	432	100	32.2	Children
Uchino M, et el.^81^	Japan	Cross sectional	561	83.5	11.6	Visual display terminal users
Verma S, et al.^76^	India	Cross sectional	100	100	74	Computer operators
Vilela MA, et al.^79^	Brazil	Cross sectional	964	100	24.7	School children
Wang L, et al.^64^	China	Cross sectional	74	80.12	74.3	Students
Wangsan K, et al.^82^	Thailand	Cross sectional	527	100	81.02	Students
Zalat MM, et al.^40^	Saudi Arabia	Cross sectional	80	100	81.3	Visual display workers
Zayed HA, et al.^69^	Egypt	Cross sectional	108	98.18	82.4	IT professionals
Zenbaba D, et al.^62^	Ethiopia	Cross sectional	416	98.6	70.43	Students

### Pooled prevalence of computer vision syndrome

**Figure 2:**
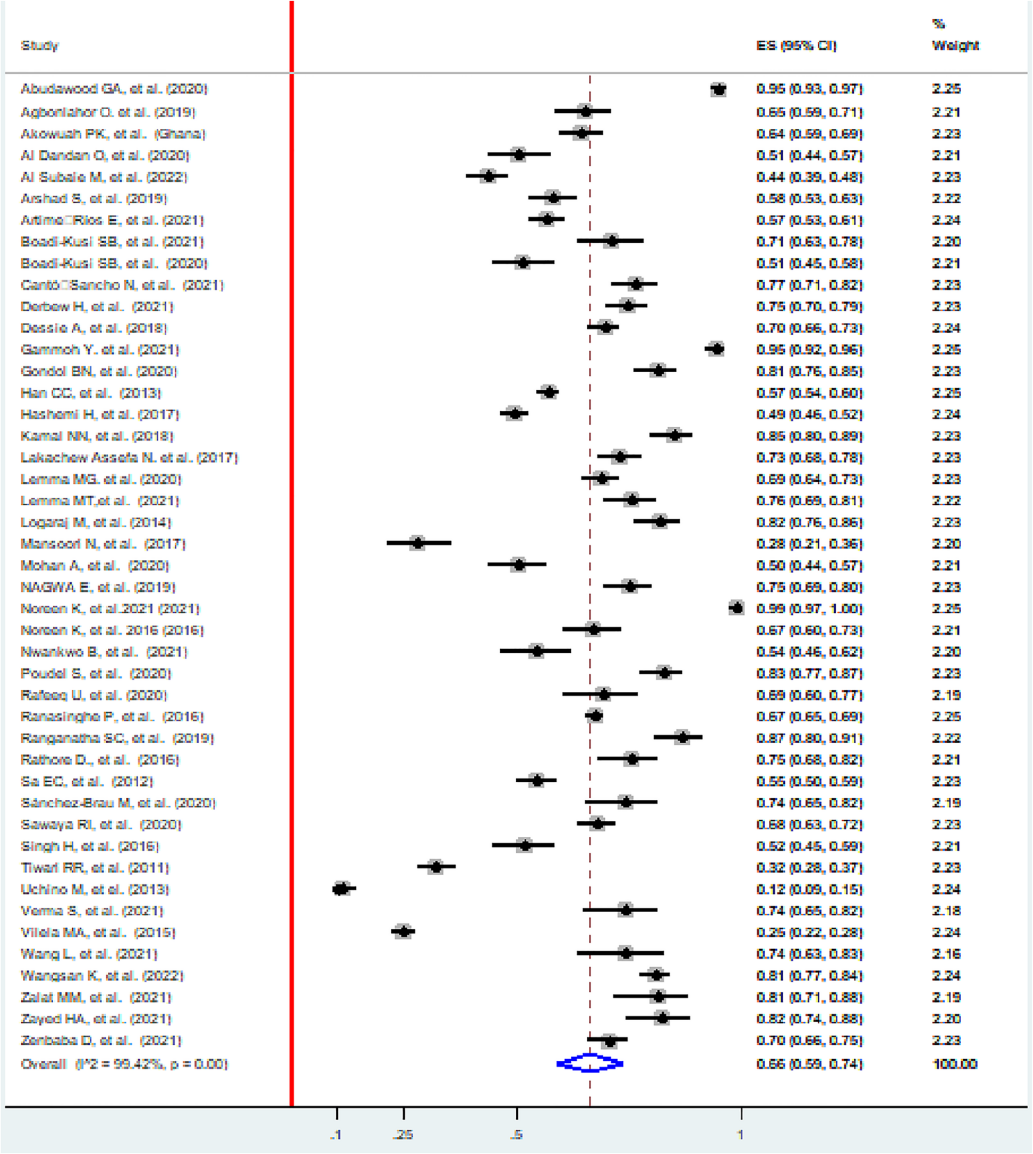
Forest plot showing the pooled prevalence of computer vision syndrome, 2022.

The pooled prevalence of computer vision syndrome was 66% (95% CI: 59, 74)**.** The lowest proportion included study was in Japan, 12% (95% CI: 9, 15)^81^, and the highest was in Pakistan, 99% (95% CI: 97, 100) ^52^. The I^2^ test showed that there was heterogeneity among the included studies (I^2^ = 99.42%, *p* value < 0.001) (Fig. 2).

### Subgroup analysis by country

Subgroup analysis was performed based on country, and the prevalence of computer vision syndrome was highest in Pakistan (97%, 95% CI: 96, 98) and lowest in Japan (12%, 95% CI: 9, 15). The studies that showed significant heterogeneity were studies in Saudi Arabia (I^2^ = 99.41%, *p* value < 0.001), Ethiopia (I^2^ = 72.6%, *p* value < 0.001), Egypt (I^2^ = 80.06%, *p* value < 0.001), and India (I^2^ = 98.04%, *p* value < 0.001) (Table 2).

**Table 2:** Subgroup analysis by country on computer vision syndrome, 2022.

Sub group		Number of included studies	Prevalence (95% CI)	Heterogeneity statistics	
				*p* value	I^2^(%)
By country	Saudi Arabia	4	68(37, 98)	*p* < 0.001	99.41
	Nigeria	2	61(56, 66)	*p* < 0.001	0.00
	Ghana	3	62(52, 73)	*p* < 0.001	0.00
	Pakistan	2	62(58, 66)	*p* < 0.001	0.00
	Spain	3	69(55, 83)	*p* < 0.001	0.00
	Ethiopia	7	73(70, 76)	*p* < 0.001	72.6
	Jordan	1	95(92, 96)	–	0.00
	China	2	58(56, 61)	*p* < 0.001	0.00
	Iran	1	49(46, 52)	–	0.00
	Egypt	5	81(74, 87)	*p* < 0.001	80.06
	India	8	65(49, 81)	*p* < 0.001	98.04
	Pakistan	2	97(96, 98)	*p* < 0.001	0.00
	Nepal	1	83(77, 87)	–	0.00
	Seri Lanka	1	67(65, 69)	–	0.00
	Brazil	2	33(30, 35)	*p* < 0.001	0.00
	Beirut	1	68(63, 72)	–	0.00
	Thailand	1	81(77,84)	–	0.00
	South Korea	1	66(63, 69)	–	0.00
	Italy	1	15(11, 21)	–	0.00
	Japan	1	12(9, 15)	–	0.00

## Meta regression

Meta-regression was performed to identify the source of heterogeneity across the studies by country and sample size. Meta-regression indicated that heterogeneity was not associated with country or sample size (*p* value > 0.05) (Supplementary file 3 Table S1).

## Publication biases

Publication bias was checked using dot plots, and visual inspection suggested asymmetry *(*Supplementary file 4: Figure S1).Moreover, publication bias was not shown by Egger’s test (*p* = 0.21) *(*Supplementary file 5 Table S2).

### Sensitivity analysis

The sensitivity analysis was performed, and no single study unduly influenced the overall effect estimate of CVS (Supplementary file 6 Table S3).

## Discussion

This systematic review and meta-analysis aimed to assess the pooled prevalence of computer vision syndrome. Although there are primary studies conducted on CVS, there are inconsistent findings on prevalence results. Moreover, there are no systematic reviews and meta-analyses on the pooled prevalence of computer vision syndrome. Therefore, findings from this systematic review and meta-analysis will help policy-makers design appropriate strategies to reduce computer vision syndrome-related public health concerns.

The pooled prevalence of computer vision syndrome was 66% (95% CI: 59, 73)**.** The pooled prevalence was in line with the study done in India COVID-19 pre lockdown, 64.3%^86^. However, the pooled prevalence was lower than that in studies performed in India during the COVID-19 lockdown, 87.3%^86^, Europe, 90%^87^, and Ethiopia, 73.21%^88^. The difference might be due to differences in study period, study setting, socioeconomic differences, awareness and behavioral change in the prevention of computer vision syndrome. Moreover, the precision of the diagnostic instruments used to record the prevalence of CVS may be the cause of a wide range of variations. Whether through direct or online surveys, the majority of papers used purely subjective questions. As most surveys rely solely on the existence of one or more CVS complaints to diagnose CVS without connecting these complaints to the time of screen use and the long-term frequency of these complaints for months, studies may exaggerate the true prevalence of CVS^11,89^. Additionally, the disparity may be caused by how people use screens, particularly smartphones, or screen abuse, such as poor lighting, uncomfortable seating positions, close eye-screen distance, improper visualization gaze, uncorrected refractive errors, prolonged continuous screen hours, a lack of breaks, viewing screens in the dark, and poor screen design.

This study has the following limitations: articles published only in English were included, and it was difficult to determine the cause-effect relationship, as all the studies were cross-sectional designs. Additional database searches, such as Science Direct, Web of Science, ProQuest, Scopus, EMBASE, etc., we’re not performed due to the lack of free access and we recommend funding to expand database searches. Moreover, this study was reported from 20 countries, which might lack representativeness.

### Conclusion

Nearly two in three participants had computer vision syndrome. Thus, preventive practice strategic activities for computer vision syndrome are important interventions.

## Supplementary Information

Supplementary Information.

## Data Availability

All data are included in this manuscript and its supplementary information files.
